# Hemoadsorption in Organ Preservation and Transplantation: A Narrative Review

**DOI:** 10.3390/life14010065

**Published:** 2023-12-29

**Authors:** Refugio García-Villegas, Stephan Arni

**Affiliations:** 1 Departamento de Fisiología, Biofísica y Neurociencias, Centro de Investigación y de Estudios Avanzados del Instituto Politécnico Nacional, D.F., Mexico City 07360, Mexico; refugio.garcia@cinvestav.mx; 2 Department of Thoracic Surgery, University Hospital Zürich, 8091 Zürich, Switzerland

**Keywords:** extracorporeal blood purification therapies, ex vivo lung perfusion, cytokine adsorption, hemoadsorption

## Abstract

Cytokine adsorption can resolve different complications characteristic of transplantation medicine, such as cytokine storm activation and blood ABO and immune incompatibilities. Cytokine adsorption is also performed for the treatment of various life-threatening conditions, such as endotoxic septic shock, acute respiratory distress syndrome, and cardiogenic shock, all potentially leading to adverse clinical outcomes during transplantation. After surgery, dysmetabolism and stress response limit successful graft survival and can lead to primary or secondary graft dysfunction. In this clinical context, and given that a major problem in transplant medicine is that the demand for organs far exceeds the supply, a technological innovation such as a hemoadsorption system could greatly contribute to increasing the number of usable organ donors. The objectives of this review are to describe the specific advantages and disadvantages of the application of cytokine adsorption in the context of transplantation and examine, before and/or after organ transplantation, the benefits of the addition of a cytokine adsorption therapy protocol.

## 1. Introduction

Cytokine adsorption (CA) is the basis of a promising therapy to resolve different complications characteristic of transplantation medicine, such as the activation of a cytokine storm, rapid immunological responses to blood incompatibilities, and immunological obstacles such as ABO blood group incompatibilities and pre-existing donor-specific antibodies. CA is performed for the treatment of various life-threatening conditions, like sepsis, acute respiratory distress syndrome (ARDS), and cardiogenic shock, all potentially worsening the transplantation clinical outcomes. After transplantation surgery, frequent dysmetabolism and stress response limit successful graft survival and can lead to primary (PGD) or secondary graft dysfunction. In this clinical context, and given that a major problem in transplant medicine is that the demand for organs far exceeds the supply, a technological innovation such as a CA system could greatly contribute to increasing the number of usable organ donors. The objectives of this review are to describe the specific advantages and disadvantages of the application of CA in the context of transplantation and examine, before and/or after organ transplantation, the benefits of the addition of a complementary CA therapy protocol.

## 2. Development of Therapeutical Cytokine Adsorption Approaches

Over the past two decades, CA therapy has progressed both in number, with the appearance of many types of adsorbers, and in application rates for new therapies. This review does not elaborate on the types of absorbers and their recommended uses. Other reviews specifically address these themes ([1] and Ankawi [2]). Indeed, Ronco reviews the application of various dialysis membranes and dialyzers in the context of persistent hemodialysis treatment and associated therapies [1]. Routinely, the composition of dialysis membranes (cellulosic or not cellulosic) and their water permeability (low flux or high flux) are the basis of their classification. The article provides an updated analysis covering new permeability indices, adsorption magnitude, the strong or poor water affinity of membranes, and electrical potential. The article also reviews basic mechanisms, such as diffusion, convection, adsorption, and ultrafiltration, in the context of therapies. The article highlights new treatments, such as expanded hemodialysis with membranes designed to generate high levels of internal filtration. They also scrutinize the clinically acceptable equilibrium between large-solute elimination and excessive albumin removal for extracorporeal therapies. Ankawi [2] describes several new adsorption cartridges, with better features developed over the years. These cartridges have expanded therapeutic applications, such as the treatment of inflammatory symptoms, chronic uremic diseases, autoimmune disorders, and poisoning. The article suggests that effective assessment of middle molecule adsorption is achieved with β2-microglobulin, α1-microglobulin, and albumin leakage assessment in the course of a dialysis session. They suggest that depletion ratios of >80% for β2-microglobulin and >35% of α1-microglobulin are beneficial to alleviate severe dialysis-related symptoms.

### 2.1. Experimental Studies

Early work by Cole evaluates an extracorporeal circuit for the removal of cytokines by coupling both large-pore hemofiltration and sorbent adsorption after incubating blood samples from six healthy volunteers with endotoxin [3]. Control blood remained at room temperature, while treatment group blood was treated ex vivo for 6 h in a closed loop through both polysulfone large-pore and activated charcoal columns. The concentrations of IL-1β, -6, -8, and -10 and TNF-α were measured at three sites (pre-hemofilter for the circulating concentration, and at both column entrance and exit). Most of the preformed circuit cytokines were removed, except for IL-10. These findings suggest that the association of a large-pore hemofilter and a charcoal column can efficiently remove a number of cytokines under ex vivo conditions. In 2010, Kakishita investigated the cytokine removal from porcine heart–lung blocks and ex vivo lung perfusion (EVLP), with or without membrane adsorption, over 12 h [4]. The study found that TNF-α and IL-8 levels were elevated in the control group perfusate after 2 h perfusion without membrane adsorption. Despite significantly reduced levels of both TNF-α and IL-8 in the membrane adsorption group, there was no observed difference in pulmonary vascular resistance, tissue oxygenation, edema formation, or myeloperoxidase activity between the two groups. The study suggests that cytokine removal using an adsorbent membrane during EVLP can suppress inflammatory cytokines, but additional factors other than cytokines may contribute to lung injury during EVLP. Pomare Montin investigated the potential in vitro cytotoxic effects of different sorbent cartridges on U937 monocytes [5]. The study evaluated the biocompatibility and cytotoxicity of four different sorbent cartridges (HA130, HA230, HA330, and HA380, Jafron Biomedical, Zhuhai City, China). After cartridge exposure under both static and dynamic conditions, the study found that the monocytes showed no signs of accelerating necrosis or apoptosis. This suggests that HA cartridges convey an excellent level of biocompatibility, with any sign of adverse reactions or cytotoxicity linked to their use in hemoperfusion (HP). In a porcine model, Fiedler evaluated the performance of two different pumpless extracorporeal hemadsorption technique (pEHAT) for CA [6]. For this purpose, two different hemoadsorber systems were used: a pEHAT system (CytoSorb^®^ (CytoSorbents, Monmouth Junction, NJ, USA)) and a commercially available EHA system (oXiris^®^ (Baxter International Inc., Deerfield, IL USA)). Both systems were connected through an arteriovenous shunt between the femoral artery and femoral vein. The mean arterial pressure (MAP) was incremented by 10 mmHg steps between 45 mmHg and 85 mmHg with the use of infusion with different drugs. The overall efficiency of the two pEHAT systems were similar and maintained sufficient blood flows extending over a large range of MAPs.

### 2.2. Clinical Studies

In 2016, Butt suggested that further research into the predictive/prognostic utility of molecular biomarkers and the development of molecular-based therapies such as CA are necessary to improve the prognoses of patients with ALI/ARDS [7]. Moreover, in severe burn patients, Rieder concluded that a veno-venous extracorporeal membrane oxygenation support and CA effectively and safely treated ALI/ARDS cases and might improve outcomes in this patient population [8]. Masakane discuss the current approaches to remove middle molecules from the blood of patients undergoing dialysis [9]. The authors call attention to the excessive adsorption of middle molecules or larger low-molecular-weight proteins as a developing concern, with harmful consequences for both the mortality and morbidity of chronic dialysis patients. They also note that when using high cut-off or medium cut-off (MCO) membranes for convective therapy during a dialysis treatment, one should avoid massive albumin leakage. In order to optimize middle molecule removal, the increase of convection volume is an important variable; a larger convection volume will increase the possibility of albumin leakage. Predilution hemodiafiltration is a useful method to avoid important albumin losses and increase the recovery of larger low-molecular-weight proteins. The article highlights the importance of assessing the efficacy of these approaches of middle molecule removal in dialysis patients to improve clinical outcomes. Calabro describes the usefulness of CA for critically ill patients with multiple organ failure (MOF) [10]. The author evaluates the clinical outcomes of CA therapy on such patients. The study includes 40 patients with cardiogenic shock, septic shock, ARDS, and liver failure. The study found that after CA treatment, total bilirubin, lactate, CPK, and LDH levels decreased significantly in critically ill patients, mainly due to cardiogenic shock. However, the study also reported a 55% thirty-day mortality rate and a 52.5% intensive care unit (ICU) mortality rate rather than the expected ICU mortality rate of 80%. Lewis observed that CA use in the ICU setting removed the mediators of inflammation, toxins, and drugs from the circulation and in combination with extracorporeal membrane oxygenation (ECMO) or other extracorporeal support systems, is purported to improve organ function and reduce morbidity and mortality in critically ill patients [11]. The effects of CA on drug molecules remain poorly understood. This presents a challenge for clinicians managing patients treated with CA, as drug concentrations may be altered, leading to potential adverse events or therapeutic failure. Supady critically reviewed the evidence regarding the clinical benefits and potential harms of extracorporeal HA [12]. The author argued that the evidence for the efficacy of HA is uncertain and that no study has shown a survival benefit. The review also points out the limitations and risks of HA, such as high costs, technical complexity, and possible adverse effects on the immune system. In addition, several potential side effects and risks associated with adsorbing devices were identified, such as bleeding, infection, and the potential for leukapheresis. Hawchar reviewed the results of the International CytoSorb^®^ Registry and showed that CytoSorb^®^ is a secure and potent therapy for the treatment of various life-threatening conditions, such as endotoxic septic shock, ARDS, and cardiogenic shock [13]. In particular, the registry results indicate that CytoSorb^®^ therapy is associated with significant trimming of ICU and hospital length of stay as well as the need for artificial ventilation and loss of life. The registry results show that the use of CA is associated with an improvement in organ function, particularly in patients with severe endotoxic septic shock and ARDS. Overall, the results of the International CytoSorb^®^ Registry demonstrate the potential of CA to improve patient outcomes and reduce healthcare costs. The registry results further suggest that CA should be considered for use in patients with life-threatening conditions, particularly those with endotoxic septic shock, ARDS, or cardiogenic shock. New research directions in the field of CA and its applications in transplantation could encompass an exploration of the modulation of the immune response beyond the immediate post-transplant period. Understanding the interactions between AC and broader immune factors, such as the associations with blood groups suggested by Alexandra [14] or associations with abnormal levels of circulating neutrophil extracellular traps suggested by Lindstedt [15], could pave the way to new therapeutic strategies.

## 3. Therapeutic Applications of Cytokine Adsorption in Transplantations

### 3.1. Therapeutic Applications in Lung Transplantation

#### 3.1.1. Experimental Studies

In 2008, Kellum determined the practicability of cytokine removal with a hemoadsorption (HA) device in a study conducted in brain-dead subjects considered unfit for organ donation [16]. The authors established that the feasibility of CA therapy did not differ from 1 h to 4 h, and the elimination fluctuated across the CA device from 4% to 30%. The study found that CA could reduce plasma concentrations of IL-6 and TNF-α, but not IL-10, in the first hour of therapy, and the effect was unsustained over time. In 2017, Iskender reported a randomized, controlled animal study in which pigs were subjected to prolonged EVLP with and without perfusate CA, [17]; see also Table 1. The experimental animals underwent evaluation of the pulmonary function and the measurement of the cytokine levels in the bronchoalveolar lavage fluid. The control group was subjected to EVLP without cytokine filtration, while the experimental group was subjected to EVLP with CA. The results of the study showed that the experimental group had significantly better lung function versus the control group, with lower levels of cytokines in the bronchoalveolar lavage fluid. This suggests that perfusate CA during prolonged EVLP is safe and effective at improving pulmonary function and reducing inflammation. In another follow-up study, in a large animal model, Iskender observed with 6 h of CA a sharp decrease of perfusate cytokine concentrations that correlated with better EVLP physiology and biochemistry [18,19,20]. This protocol improved both transplanted lung dynamic compliance and oxygenation function in the treated group after 4 h of reperfusion and decreased local inflammatory response. These findings suggest that the EVLP protocol was improved after implementing a CA in the circuit. This study provides evidence that perfusate CA during EVLP can improve short-term graft function after LuTx and may be an effective strategy to recondition the allograft after ischemia reperfusion-induced injury (IRI) and may improve patient outcomes following LuTx.

In a large animal model, Frick uses a porcine-LuTx model and finds that continuous CA results in a significant improvement in lung graft function, as assessed by the degree of allograft injury, post-transplant survival, and blood chemistries [21]. The study also reports a significant reduction in pro-inflammatory cytokine and chemokine levels in the systemic circulation of the animals. These results suggest that CA may attenuate IRI in the allograft after LuTx. Furthermore, the findings suggest that the inflammatory cytokines and chemokines play an important role in the pathogenesis of IRI. In a large animal model, Niroomand investigates how CA affects lung function both during EVLP and post-transplant. The study hypothesis states that the inflammatory and immunomodulatory differences in lungs treated with or without CA contribute by modulating the molecular mechanisms and signaling pathways that impact lung function [22]. The results show that, after CA treatment, both inflammatory and immune processes and coagulation pathways are affected significantly. Moreover, Niroomand also reviews the EVLP technology that was developed as a therapeutic platform to improve donor lung quality prior to lung transplantation (LuTx) [23]. The application of EVLP as a therapeutic intervention has led to significant improvements in donor lung quality and has allowed for the successful transplantation of organs previously considered too damaged or too marginal to be used. EVLP reduces inflammation, ameliorates lung function, and avoids post-transplant complication risk. Additionally, EVLP has been used to evaluate the efficacy of various cell therapies and cell product therapies, as well as to test the effectiveness of CA. In a large animal model, Ghaidan shows that the two-step treatment with CA is effective in improving lung function in ARDS, as indicated by a significant increase in the PaO_2_/FiO_2_ ratio [24]. Furthermore, this treatment was associated with a reduction in the incidence of PGD, a common severe complication associated with LuTx that affects up to 50% of recipients and increases the risk of chronic rejection and mortality. Molecular outcomes showed that the two-step treatment resulted in decreased systemic inflammation and oxidative stress in the lungs and other organs. The treatment was accompanied by improved tissue oxygenation and reduced cellular damage. In addition, the two-step treatment was associated with a decrease in the expression of inflammatory genes and a decrease in the number of inflammatory cells. Taken together, these results suggest that the two-step treatment with the CA is effective in improving lung function and reducing the incidence of PGD with ARDS after LuTx. In the same context, Ehrsam reports the applicability of CA during and after reperfusion as a viable approach to reduce post-transplant inflammation following LuTx [25]. The study was conducted on pig left LuTx previously exposed to 24 h of cold ischemic storage. The control group did not receive extracorporeal CA, whereas the treatment group received adsorption for 6 h post- and 30 min pre-reperfusion. A significant reduction in plasma pro-inflammatory IL-2 was noticed during CA, along with trends of lower pro-inflammatory TNFα, IL-1α, and GM-CSF and significantly fewer systemic neutrophils. The study found that CA during and after reperfusion is effective in reducing post-transplant inflammation following LuTx. Compared to the control group, the treatment group exhibited significant improvement in CO_2_ elimination, in lessening of acidosis, and in the PaO_2_/FiO_2_ ratio. The study concludes that CA during and after reperfusion is a feasible technique to lessen post-transplant inflammation following LuTx.

#### 3.1.2. Clinical Studies

In human patients, Peyneau performed a small-sample-size study with a single-arm pilot design that was limited by the absence of a control group, making it difficult to draw conclusions about the efficacy of CA therapy compared to standard treatment [26]. The study also had a short follow-up period, and the long-term effects of CA therapy remain unchartered. Despite these limitations, the results of this study provide important preliminary evidence regarding the potential of CA therapy to reduce inflammation and improve clinical outcomes in LuTx. Moreover, Boffini describes the value of EVLP to remove cytokines from lungs prior to transplantation [27]. Among the 54 EVLPs performed, 21 were handled with and 33 without CA therapy. At the end of the process, the authors observed a significant decrease in IL-10 and GCSF in the CA-treated group. After the EVLP performed with CA, they recorded a significant decrease in the amount of IL-6 and GCSF, but also IL-10 and MCP1, as determined from the transplanted patients. This transplanted group also had lower in-hospital mortality (*p* = 0.03) and a lower 1-year death rate (*p* = 0.01). The results of this human study suggest the value of CA and EVLP to effectively decrease the concentration of inflammatory mediators, indicating both the safety and effectiveness of this procedure. Even if more research is needed to fully understand the clinical impact of cytokine reduction during EVLP, this study found that EVLP with cytokine removal was safe and feasible and that it could be used to improve the quality of lungs for transplantation. Lindstedt discusses the applicability of CA during LuTx to reduce or remove elevated levels of neutrophil extracellular traps (NETs) [15]. NETs are composed of a network of neutrophil DNA associated with microorganisms and form an important antimicrobial mechanism of neutrophilic granulocytes. NETs modulate both inflammatory and autoimmune diseases and are involved in organ transplantation. NETs can induce tissue damage, activate the complement system, promote thrombosis, and trigger adaptive immune responses. In LuTx, NETs have been associated with PGD. NETs can impair alveolar gas exchange, increase vascular permeability, and induce inflammation and fibrosis in the transplanted lung. The study found that using a CA adsorber during LuTx may diminish the systemic inflammatory state by filtering out NETs and consequently reinforcing graft acceptance. The CA-treated group of patients had fewer circulating nucleosomes and at 30 and 90 days post-LuTx remained clear of PGD and indications of acute rejection in comparison to the patient group without CA. The study’s findings suggest that CA could be a method for clearing NETs. Lindstedt describes a Swedish national interventional randomized controlled study (NCT05242289) involving 116 patients [28]. The study investigates the potential benefits of a cytokine filtration administered for 12 h in the first 24 h following LuTx that may improve graft outcomes. The clinical trial objectives were to demonstrate the advantage of CA in improving LuTx success, as a consequence of its effects on oxygenation index, plasma levels of inflammatory markers, PGD incidence and severity, lung function, kidney function, survival, and quality of life compared with standard treatment without cytokine filtration. Previous research [24] found that cytokine filtration can modulate pulmonary metabolism and edema formation during EVLP. The process involved exposure of the graft to a 24 h ischemic time at 4 °C, followed by an EVLP of 12 h, where the perfusate of the treated group was continuously run through CA. This procedure resulted in significant improvement in airway pressure and dynamic compliance, with reduced glucose utilization and lactate accumulation in the CA group. This suggests that CA could potentially improve outcomes in LuTx.

In conclusion, and also listed in Table 1 (see column “Effects of CA therapy”), much experimental and clinical evidence at different interventional steps (pre-, during, post-) of lung transplantation support the notion that CA therapy indeed improves the functional graft outcomes (↑ oxygenation, ↑ compliance, ↓ pharmacotherapy, ↑ CO_2_ removal, ↓ neutrophil extracellular traps, ↓ coagulation and complement pathways, ↓ humoral immune response pathways) and tends to lessen early pathological complications (↓ in-hospital mortality, ↓ PGD, ↓ AR).

**Table 1:** Effect of CA therapy on different models involving large, solid organs and transplantations.

Organ Targeted	Model and Treatment	Effects of CA Therapy	References
Lung	Porcine EVLP + CA, then LuTx.	↓ cytokines, ↑ oxygenation, ↑ compliance, ↓ pharmacotherapy.	[18,19,20]
	Porcine EVLP + CA, then LuTx.	↓ coagulation and complement pathways, ↓ humoral immune response pathways.	[22]
	Porcine EVLP + CA.	↓ pulmonary oedema, ↓ electrolytes imbalance.	[17]
	Porcine EVLP + CA, then LuTx + CA.	↓ cytokines, ↓ immune cells, ↓ PGD.	[24]
	Porcine post LuTx and CA.	↓ cytokines, ↑ CO_2_ removal.	[25]
	Human LuTx and CA.	↓ neutrophil and monocyte activation markers.	[26]
	Human EVLP + CA, then LuTx.	↓ cytokines, ↓ in-hospital mortality.	[27]
	Human intraoperative LuTx and CA.	↓ neutrophil extracellular traps, ↓ AR and PGD.	[15]
Heart	Human CPB + CA.	↓ hemodynamic and metabolic and organ instability.	[29,30]
	Human CPB + CA.	↓ plasma-free hemoglobin and complement C3a and C5a.	[31]
	Human CPB + CA.	↑ IL-10 anti-inflammatory long-lasting effects.	[32]
	Human CPB for infective endocarditis + CA.	↓ cytokines but without resolution of hemodynamic instability and in-hospital mortality.	[33]
	Human CBP for acute endocarditis + CA.	↓ in vasopressor use but not significant.	[34]
	Human intraoperative HTx and CA.	↓ CRRT frequency.	[35]
	Human donor heart resuscitation for HTx and CA.	Donor heart implantation after 7 h cold ischemia.	[36]
	Human HTx and CPB and CA.	Control of heparin-induced thrombocytopenia in HTx.	[37]
	Human VA-ECMO for giant-cell myocarditis and CA.	↓ hemodynamic and metabolic and organ instability.	[38]
	Human VA-ECMO for cardiogenic shock and CA.	↓ lactate, ↑ urine output, ↓ in-hospital mortality.	[39]
	Porcine DCD heart ex vivo perfusion and CA.	↓ cytokines, ↓ markers of endothelial injury.	[40]
Kidney	Porcine kidney ex vivo perfusion and CA, then KTx.	↑ IL-6 and IL-8 at reperfusion, ↑ mean renal blood flow, ↓ prostaglandin E2 and prostacyclin and thromboxane.	[41]
	Human FSGS + CA.	↓ soluble urokinase plasminogen activator receptor.	[42]
	Human FSGS + lipoprotein apheresis.	↑ complete or partial remission of proteinuria, ↑ response rates to steroid or immunosuppressive therapy.	[43]
	Human kidney ex vivo perfusion and CA, then KTx.	↑ oxidative phosphorylation (OXPHOS), ↓ inflammatory pathway genes.	[44]
	Human FSGS + CA.	↓ proteinuria.	[45]
Liver	Human ALF + CA.	↓ TNFα and CRP and procalcitonin and α1-fetoprotein.	[46]
	Human ACLF + CA.	↓ TNFα, ↑ IL-6.	[47]
	Human LF + CA.	↓ TNFα, ↓ IL-6, ↓ IL-1, ↓ IL-10.	[48]
	Human LF + CA.	↓ bilirubin, ↓ ammonia, ↓ LDH, ↓ platelets.	[49]
	Human LF + CA.	↓ bilirubin.	[50]
	Human hyperbilirubinemia + CA.	↓ bilirubin.	[51]
	Human ALF + CA.	↓ bilirubin.	[52]
	Human septic shock after LiTx + CA.	↓ procalcitonin, ↓ endoxins, ↓ IL-6, ↓ IL-10.	[53]
	Human hyperbilirubinemia + CA.	↓ bilirubin.	[54]
	Human ACLF + CA.	↓ bilirubin.	[55]
	Human ACLF + CA.	↓ bilirubin.	[56]
	Human hyperbilirubinemia + CA.	No change in 30-day hospital mortality.	[57]
	Human pediatric hyperbilirubinemia + CA.	↓ bilirubin.	[58]
	Porcine DCD liver ex vivo perfusion and CA.	↓ cytokines.	[59]

### 3.2. Therapeutic Applications in Heart Transplantation

#### 3.2.1. Experimental Studies

In a large animal model, Saemann used a normothermic donation after circulatory death (DCD) heart model, in which the DCD hearts were perfused with the donor’s blood collected before and after CA [40]. The study found that CA during blood perfusion (BP) of DCD hearts significantly reduced coronary microvascular dysfunction, oxidative stress, and IRI of the coronary microvascular endothelium compared to hearts perfused without CA. The author also observed that CA during BP was associated with a reduction in the expression of the proinflammatory genes TNF-α, IL-1β, IL-6, and IL-8 and a significant overexpression of the anti-inflammatory genes IL-10 and TGF-β. The results suggest that CA during BP of DCD hearts may be beneficial in reducing coronary microvascular dysfunction, oxidative stress, and IRI of the coronary microvascular endothelium.

#### 3.2.2. Clinical Studies

Early application of CA in the management of heart diseases was investigated by Trager [29]; see also Table 1. The author used a CA device in order to control hyperinflammatory systemic reactions in patients undergoing cardiothoracic surgery with cardiopulmonary bypass (CPB). This retrospective case series involved 16 cardiac surgery patients who developed severe inflammatory response syndrome (SIRS) and subsequent AKI following prolonged CPB. The treatment reduced cytokine concentrations, and this reduction was associated with a rectification of disturbed hemodynamic, metabolic, and heart function variables. The safety and tolerance of the treatment was high, without showing any CA-related adverse complications. Moreover, Trager also used CA during CPB as a way to reduce the pro-inflammatory response and improve postoperative organ function [30]. The reduction in the severity of postoperative organ dysfunction was explored in a clinical trial investigating the value of CA during surgery. The usefulness of CA for patients during CPB surgery due to infective endocarditis was also investigated, showing a decline of pro-inflammatory cytokines. Furthermore, the assistance of CA in DCD hearts during BP has been suggested to reduce the pro-inflammatory response, vascular damage, and IRI of the coronary microvascular endothelium, which may ultimately improve graft survival. In 2018, Nemeth proposed a CA treatment in the course of HTx surgery that reduced the need for (1) vasopressor requirement, (2) continuous renal replacement therapy (CRRT), and (3) lengthy mechanical ventilation and ICU stay [35]. This suggests that CA may be a useful adjunct to improve outcomes in patients undergoing orthotopic HTx. Dogan describes the successful use of CA in combination with extracorporeal life support therapy in a patient with giant-cell myocarditis [38]. Large and sustained improvements were recorded in both hemodynamic and inflammatory parameters with the resolution of metabolic acidosis to normal values and improved liver function. This case report highlights the potential of CA to improve organ function and inflammation following the extracorporeal support of a patient with giant-cell myocarditis and may represent an additional therapeutic option for patients with a severe form of this disease. In the context of prolonged cardiovascular bypass, Gleason reports the results on the safety and potentiality of CA therapy in a prospective, multicenter REFRESH (REduction in FREe Hemoglobin) clinical trial that assesses the reduction of plasma-free hemoglobin and activated complements during prolonged CPB in patients undergoing elective, nonemergency complex cardiac surgery [31]. Enrolled patients were allocated to either obtain CA therapy or standard medical therapy (SMT). The pilot study found that CA therapy during complex cardiac surgery was safe and achievable. This CA pilot study recorded significant decreases in both plasma-free hemoglobin and C3a and C5a levels. Bernardi also investigated the effect of CA therapy during CPB surgery and hypothesized that the CA of cytokines may suppress inflammatory responses and improve patients’ perioperative course [32]. Of the 37 patients undergoing elective CPB surgery, a total of 19 were randomly assigned to CA therapy and 18 to the control group. The authors found that there were no differences in cytokine concentrations of IL-1β, IL-6, IL-18, TNF-α, and IL-10 as primary outcomes immediately following the CA treatment. Nonetheless, they observed a long-lasting anti-inflammatory effect of IL-10. In a case report, Kaliyev describes the clinical data of an HTx performed after 7 h in cold Custodiol solution and paracorporeal donor heart resuscitation [36]. The authors used controlled warm reperfusion, medical treatment, and CA to resuscitate the donor organ. The report also mentions that “suboptimal” organs are transplanted more often, and this approach could potentially increase the viability of organs for transplantation and improve patient outcomes. Diab conducted a trial in two phases [33]. In the run-in phase, all patients underwent conventional cardiac surgery for infective endocarditis, and intraoperative CA was performed in a subset of patients. Patients in the CA group had significantly lower plasma levels of inflammatory mediators than those in the control group. In the randomized phase, patients were assigned to the CA or control group and were assessed for changes in Sequential Organ Failure Assessment (SOFA) score from postoperative day 0 to day 3. Secondary outcomes included postoperative infection, extent of stay in the ICU, and mortality. The results showed that patients in the CA group had significantly lower SOFA scores at postoperative day 3 than those in the control group, but no significant differences in the matter of postoperative infection, extent of stay in the intensive care unit, or mortality were found. As measured by changes in the SOFA score, these findings suggest that intraoperative CA may be beneficial for patients experiencing cardiac surgery for infective endocarditis. In a single-center pilot study, Poli studied CA therapy in patients undergoing elective cardiac surgery [60]. The patients were randomly distributed in two groups of 15 and treated either with or without CA therapy. The authors concluded that CA therapy did not improve clinical outcome and did not ameliorate the levels of pro- or anti-inflammatory cytokines. In 2022, Holmen reported that CA during cardiac surgery may reduce the need for vasopressors after surgery for endocarditis, but despite a decrease both in the need for red blood cell transfusion and in the dosage of norepinephrine in the treated group, at all time points, those differences did not reach statistical significance [34]. Overall, these results indicate that CA during cardiac surgery may reduce the need for vasopressors after surgery for endocarditis. In 2023, Lovric presented several findings related to the applicability of CA in patients with signs of cardiogenic shock and treated with veno-arterial extracorporeal membrane oxygenation (VA-ECMO) [39]. From the 16 patients included in the study, and stratified based on CA application in the first 24 h of treatment, the patients treated with CA required significantly lower doses of vasopressors at the start and before weaning from VA-ECMO, with significantly greater urine output ahead of weaning and lower lactate levels during VA-ECMO. The mortality rate was lower among the group that received CA therapy (22.2% vs. 57.1%). This study suggests that CA can lead to improved urinary output and a tendency for better survival among VA ECMO patients. In conclusion, and also listed in Table 1 (see column “Effects of CA therapy”), many clinical and some experimental results at different interventional steps (pre-, during, post-) of heart transplantation support the notion that CA therapy may improve, in some cases, the functional graft outcome (↓ hemodynamic and metabolic and organ instability) and may tend to lessen early pathological complications (↓ in-hospital mortality, ↓ markers of endothelial injury).

### 3.3. Therapeutic Applications in Kidney Transplantation

#### 3.3.1. Experimental Studies

Early application of CA in the management of kidney diseases was investigated in a large animal model by Hosgood [41]; see also Table 1. The author studied the effect of incorporating CA into an isolated kidney perfusion system. The results showed that during perfusion, CA removed inflammatory mediators and functionally improved kidney blood flow.

#### 3.3.2. Clinical Studies

In 2017, Schenk investigated the therapeutic effects of soluble podocyte urokinase receptor elimination through CA and total plasma exchange (PE) in the treatment of primary focal segmental glomerulosclerosis (FSGS) and recurrence after kidney transplantation (KTx) [42]. Furthermore, the study explored the exact pathophysiological role of the soluble podocyte urokinase receptor in FSGS and other kidney diseases and assessed whether soluble podocyte urokinase receptor elimination can effectively improve patient outcomes. Concerning FSGS, Raina suggests that lipoprotein apheresis is a secure and potent treatment for drug-resistant (corticosteroids and/or calcineurin inhibitors) primary FSGS and post-renal transplant primary FSGS reoccurrence [43]. The findings also suggest that lipoprotein apheresis in primary FSGS patients may be an effective treatment for inducing a temporary or permanent decrease in proteinuria symptoms. In this same context of FSGS, Karkar discusses several aspects of CRRT indication in critically ill patients with Acute Kidney Injury (AKI), endotoxic septic shock, and MOF in the ICU [61]. The aspects covered include the option of CRRT versus intermittent and extended HD. The life of the filtering/dialyzing device can be influenced by factors such as the filtration fraction and the type of anticoagulation used, anticoagulation including regional citrate anticoagulation to prevent clotting in the circuit, the prescribed versus delivered CRRT dose, and the vascular access management, which impact both the efficacy of treatment and the risk of complications. Moreover, the influence of the timing of CRRT initiation and termination is covered. In a case study, Rybalko successfully treated a patient with a complex medical condition with a combination of ECMO and multiple extracorporeal blood purification methods, including PE, CA, and lipopolysaccharide adsorption [62]. This report demonstrates the potential benefits of a multimodal approach to extracorporeal therapies in the setting of immunosuppression therapy. It also highlights the importance of careful monitoring for potential complications, such as occult infections, which may not be immediately apparent. Based on the successful outcome in this case, multimodal extracorporeal therapies may be a viable option for treating complex medical conditions in pediatric patients. Using a molecular approach, Ferdinand studied kidney transcriptomes and compared the effects of a 120 min normothermic machine perfusion (NMP) with those of a standard preservation at 4 °C [44]. The latter caused a marked reduction in the expression of genes involved in inflammatory pathways, particularly those for oxidative phosphorylation, whereas during NMP these genes, as well as those for immune processes, were regulated positively. Following this NMP, the biopsies of the transplanted grafts showed higher expression of inflammatory genes in organs with prolonged delayed graft function. Using CA to eliminate pro-inflammatory cytokines significantly reduced the cytokines in the perfusate but also reduced the expression of inflammatory genes and accelerated the expression of genes involved in oxidative phosphorylation. These effects were beneficial for the graft by decreasing the expression of a delayed genetic signature associated with graft function, potentially improving outcomes for transplant recipients. In a case study, Muller-Deile reported the success of CA as applied to a KTx patient with recurrent FSGS and a complete elimination of deleterious circulating factors [45]. The researchers previously created a model of proteinuria in zebrafish that allowed them to characterize a functional phenotype of the disease, which affects podocytes. The metabolome analysis detected lipid profiles in FSGS serum, and these profiles could reflect a new subtype of the disease. These findings provide valuable insights into the metabolic changes associated with recurrent FSGS, potentially leading to improved diagnostic and therapeutic techniques. Reis reviews types of dialysis membranes, such as high-retention-onset membranes with wider pores and a more uniform pore size distribution, allowing for the efficient removal of middle molecules such as uremic toxins and inflammatory mediators [63]. As such, high-retention-onset dialyzers are now commonly used for patients with AKI requiring continuous kidney replacement therapy, as well as those with myeloma requiring hemodialysis (HD), for free light chain removal. Other dialysis membranes reviewed are MCO membranes, which are used for maintenance in HD patients. Numerous clinical trials have demonstrated the superior efficacy of MCO dialyzers in removing uremic toxins and other middle molecules compared to traditional high-flux membranes. In a case report, Moresco describes the successful use of CA in a patient with rhabdomyolysis and kidney dysfunction following multiple sport traumas and consecutive surgical resections [64]. For this patient, the CA therapy reduced both the creatine kinase and myoglobin levels and allowed full recovery without any further complications. In conclusion, and also listed in Table 1 (see column “Effects of CA therapy”), many clinical and some experimental results at different interventional steps (pre-, during, post-) of kidney transplantation support the notion that CA therapy may improve, in some cases, the functional graft outcome (↓ proteinuria, ↑ oxidative phosphorylation (OXPHOS),); however, little evidence is reported regarding changes in early pathological complications (↑ response rates to steroid or immunosuppressive therapies).

### 3.4. Therapeutic Applications in Liver Transplantation

#### 3.4.1. Experimental Studies

In a large animal model, Ghinolfi reported a porcine liver graft obtained after circulatory death and treated with CA in an ex vivo perfusion device [59]. The authors compared, without technical failures, normo- and hypo-thermal perfusion approaches and measured the CA therapy adsorption capacity of both bile and inflammatory cytokines.

#### 3.4.2. Clinical Studies

Early approaches with plasma fractionation impacting patients with severe liver failure were investigated by Santoro [65]. Here, the PROMETHEUS^®^ (Fresenius Medical Care AG, Bad Homburg, Germany) system, which was a novel device previously characterized to collect albumin-bound toxins, was positively compared to the Molecular Adsorbent Recycling System (MARS^®^; Gambro-Baxter, Deerfield, IL, USA) in terms of elimination of albumin-bound and water-soluble substances. These findings are significant, as they provide insights into the potential therapeutic benefits of PROMETHEUS^®^ (Fresenius Medical Care AG, Bad Homburg, Germany) in managing toxin levels in patients with severe liver failure. The system works by separating plasma through an albumin-porous membrane and two adsorbers (a neutral and an anion exchanger sorbent, both removing toxin-bound proteins) and finally through a high-flux hemodialyzer that prevents water soluble substances from returning to the venous line. The authors included 12 patients in their trial with acute or acute-on-chronic liver failure (ACLF); the PROMETHEUS^®^ (Fresenius Medical Care AG, Bad Homburg, Germany) system was used to treat hyperbilirubinemia, hypercholemia, and hyperammonemia. The study found that the mean total bilirubin decreased, while the reduction for cholic acid and ammonia were up to 50%, with a significant decrease in the blood concentration of both soluble IL-2 receptor and IL-6. In 2010, Rocen discussed a study involving 11 patients with ALF and the modulation by the PROMETHEUS^®^ (Fresenius Medical Care AG, Bad Homburg, Germany) system of both cytokines and different post-signs of inflammation and liver regeneration, [46]; see also Table 1. After treatment, the blood levels of TNF-α, CRP, procalcitonin, and α1-fetoprotein decreased significantly, while those of hepatic growth factor increased, which seems to indicate the positive effects of the PROMETHEUS^®^ (Fresenius Medical Care AG, Bad Homburg, Germany) system, which resulted in reduced levels of inflammation and increased levels of factors improving regeneration. The MARS^®^ (Gambro-Baxter, Deerfield, IL USA) is another early blood detoxification system based on albumin dialysis. The impact of MARS^®^ (Gambro-Baxter International Inc., Deerfield, IL USA)therapy on cytokine metabolism in patients affected with ACLF was discussed by Ambrosino [47]. The authors selected two cohorts of patients (with either acute liver injury or graft dysfunction) and compared them using two types of interventions, the SMT or the MARS^®^ (Gambro-Baxter International Inc., Deerfield, IL USA)therapeutic system. The key findings from the study are an increase in IL-6 levels observed during MARS^®^ (Gambro-Baxter International Inc., Deerfield, IL USA)treatment and a decrease in TNF-α levels noted during MARS^®^ (Gambro-Baxter International Inc., Deerfield, IL USA)treatment. These findings are significant as they provide insights into the potential therapeutic benefits of MARS^®^ (Gambro-Baxter International Inc., Deerfield, IL USA)in managing cytokine levels in patients with acute-on-chronic liver decompensation. In 2009, Novelli discussed the impact of MARS^®^ (Gambro-Baxter International Inc., Deerfield, IL USA)on cytokine levels in patients [48]. The author conducted a clinical study to compare the effects of MARS^®^ (Gambro-Baxter International Inc., Deerfield, IL USA)therapy and SMT on cytokine metabolism and survival in 30 patients with ACLF. They found that the MARS^®^ (Gambro-Baxter International Inc., Deerfield, IL USA)therapeutic system significantly altered the levels of numerous cytokines as well as increased the hepatocyte growth factor, which may indicate enhanced liver regeneration. They also reported that MARS^®^ (Gambro-Baxter International Inc., Deerfield, IL USA)therapy improved the 3-month survival rate from 30% to 60% compared to SMT. The authors conclude that the MARS^®^ (Gambro-Baxter International Inc., Deerfield, IL USA)therapeutic system improved the physiological and pathological values of ACLF patients, ameliorating their physiological and pathological recovery, and can be used to improve spontaneous regeneration or as a link before transplantation. Popescu presents a comparison between two liver assist devices, the MARS^®^ (Gambro-Baxter International Inc., Deerfield, IL USA) and CytoSorb^®^ (CytoSorbents, Monmouth Junction, NJ, USA), in patients with liver failure [49]. Although both systems reduced bilirubin and ammonia, only the reduction of lactate, bilirubin, ammonia, and lactate dehydrogenase by the CytoSorb^®^ (CytoSorbents, Monmouth Junction, NJ, USA) system significantly improved patients’ liver tests and improved their “Model for End-Stage Liver Disease” score. In a clinical trial, Trautman selected a cohort of 49 patients with fulminant liver failure and studied the impact of the MARS^®^ (Gambro-Baxter International Inc., Deerfield, IL USA)therapeutic system on their renal outcomes [66]. Treatment of hepatic encephalopathy was the most common condition for initiating sessions with the MARS^®^ (Gambro-Baxter International Inc., Deerfield, IL USA)system. Among these patients, 29 required CKRT therapy, which ultimately caused a mortality of 41% compared to only 10% in patients who did not require CKRT therapy, with a lower one-year mortality (25%) compared to 59% for those requiring CKRT. Using a network meta-analysis in patients with ACLF, Ocskay compared different hepatic support systems with SMT [67]. A total of 16 studies with different therapeutic systems are compared (MARS^®^ (Gambro-Baxter International Inc., Deerfield, IL USA),PROMETHEUS^®^ (Fresenius Medical Care AG, Bad Homburg, Germany), ELAD^®^ (Vital Therapies, San Diego, CA, USA), PE (PE), and BioLogic-DT^®^ system (HemoCleanse, Lafayette, IN, USA). Overall survival was significantly improved by PE when compared to SMT, hence its place at the top of the cumulative ranking for overall survival (SUCRA: 86% at 3 months; 77% at 1 month) and for 3-month transplantation-free survival (TFS) (SUCRA: 87%); PE was second after ELAD^®^ (Vital Therapies, San Diego, CA, USA), for the 1-month measurement of TFS (SUCRA: 76%). This suggests that for ACLF, the hepatic support therapy is excellent for 3-month overall survival. Regarding coupled plasma filtration adsorption (CPFA), Maggi discusses CPFA usefulness in managing hyperbilirubinemia after liver transplantation (LiTx) [54]. CPFA is a therapeutic system that first filtrates plasma; then, a CA system captures cytokines and mediators of inflammation. The study reports two cases of patients who experienced different complications after LiTx. The first transplantation ended up with early allograft dysfunction, whereas the second transplantation had hyperbilirubinemia due to chronic rejection. Following three cycles of CPFA, the bilirubin levels promptly decreased in both cases by approximately 40%. This suggests that CPFA could be an effective treatment for managing hyperbilirubinemia after LiTx. In a clinical study, Donati discusses the assistance of CPFA in managing acute liver failure (LF) or ACLF [55]. The study enrolled 12 patients with acute LF or ACLF in a prospective observational study to evaluate the effectiveness of CPFA in liver detoxification. Bilirubin and bile acid reduction ratios per session were around 30% for total, direct, or indirect bilirubin, and bile acids. One patient received LiTx, one died, and eight out of nine survived 1 year of follow-up time. This suggests that CPFA could be an effective treatment for managing such conditions, and CPFA may be develop as a bridge to transplant therapy and to restore basal liver function. Following kidney and liver failures, toxin build-up occurs; in this context, Wu reviewed the history of HP and evaluated the performance of common materials such as activated carbon, inorganic porous materials, and polymers regarding their constitution, their toxin adsorption abilities and mechanisms, and their biocompatibility and blood harmlessness [68]. Regarding the Double Plasma Molecular Adsorption System (DPMAS), Rosa-Diez discusses the application in relation to liver failure in the ICU, whether acute or ACLF [69]. DPMAS is a nonbiological artificial liver support system that combines two types of resins: a ion exchange and a neutral macromolecule adsorber. These adsorbents can selectively remove bilirubin, cytokines, and other medium- and macro-molecular toxins from plasma. The authors also review the development of a new class of HP adsorbents that can improve the treatment of usual blood in hepatic and renal failure. Moreover, Marcello discusses the application of the DPMAS therapeutic system to adsorb bilirubin following loss of liver function during ALF and ACLF [70]. Hyperbilirubinemia is prognostically unfavorable for patients with endotoxic septic shock or MOF who are at risk of developing ALF. Elimination of bilirubin not only may alleviate signs and symptoms of liver dysfunction but also may serve as an index of elimination of albumin-related toxins. Conjugated and unconjugated bilirubin, due to their molecular weight and albumin-binding capacity, respectively, are unremoved by conventional dialysis. However, DPMAS is a therapy that supports liver function using a broad adsorption technique that reduces the concentration of inflammatory mediators and toxins such as bilirubin. Interestingly, Marcello [56] also presents a new cartridge (BS330, Jafron Biomedical, Zhuhai City, China) for adsorbing hyperbilirubinemic plasma. The author observed a rapid reduction of 16.5% in plasma bilirubin concentration at 30 min as well as a retention of 759 mg after 2 h, with a total calculated retention capacity of 5.76 mg of bilirubin per gram of resin. Regarding CA therapy, Acar concludes that it is an effective and safe alternative treatment for hyperbilirubinemia associated with sepsis in patients with liver failure [50]. The CA therapy can reduce both bilirubin levels and vasoactive medication requirements. Additionally, one should consider the inability of CA therapy to reduce ammonia levels when planning treatment for patients with liver failure and endotoxic septic shock. Moreover, Tomescu demonstrates the clinical efficacy of CA in ALF, with an improvement in liver functional tests and a decrease in CRP title [71]. In addition, Ocskay highlights that extracorporeal HA with CA appears to be a promising treatment for acute forms of liver dysfunction and failure [72]. The CA therapy effectively removes bilirubin, bile acids, and ammonia from the blood. In a case report, Rachunek highlights the potential of CA as a therapeutic tool to improve wound healing in patients with severe burns and liver dysfunction [51]. The CA therapy was successful in reducing the bilirubin concentrations and allowing for successful wound healing, despite the patient’s underlying secondary sclerosing cholangitis. The patient was able to recover from the burns and successfully undergo skin grafting to close the wounds. Potentially, in severely burned patients with altered liver function, the CA therapy could mitigate the risks of infection and assist in wound healing. In addition, Hui argues that extracorporeal blood purification with CA is an effective and safe modality for bilirubin removal among patients aged 1 month to 18 years [58]. Per session, the bilirubin removal ratio was 44.6% with interquartile range of 14.5%. The patients who received LiTx all showed significant improvement in hepatic enzymes without important procedure-specific complications. Despite any CA therapy-related mortality, the overall pediatric intensive care unit cohort mortality was 50%. Interestingly, Grafe investigated whether the extracorporeal elimination of bilirubin with a CA reduces mortality in patients with hyperbilirubinemia [57]. The study included patients with bilirubin concentrations >10 mg/dL and who were undergoing kidney replacement therapy in the ICU. The results showed no significant differences in patients with and without CA treatment. The multivariate model showed no significant effect of CA therapy (*p* = 0.402) on 30-day mortality. In addition, a significant effect of bilirubin concentration (*p* = 0.274) or Model for End-Stage Liver Disease score (*p* = 0.928) on the 30-day mortality could not be shown. In contrast, lactate concentration (*p* = 0.001, b = 0.044) and SAPS II (*p* = 0.025, b = 0.008) had a significant impact on 30-day mortality. They conclude that CA in hyperbilirubinemic patients was not significantly associated with a reduction in the 30-day mortality. In a case report, Frimmel demonstrates the combined use of single-pass albumin dialysis with CA in a patient with ALF and most likely with SIRS hemophagocytic lymphohistiocytosis [52]. The study found that the 12 h CA therapy reduced the IL-6 concentration from 81,059 pg/mL to 17,177 pg/mL, and this CA therapy allowed reduction of norepinephrine dosage to 0.25 mg/kg/minute. CA therapy was deemed safe and acceptable by the patient, without any adverse events. This suggests that the combined use of single-pass albumin dialysis with CA could be an effective treatment for managing such conditions. In a case report, Li describes CA therapy with CRRT for the control of septic shock of a 35-year-old liver-transplanted patient [53]. The treatment included antibiotics, antifungals, and antivirals in addition to the CA-CRRT therapy. After 2 days of CA-CRRT, the blood pressure of the patient ameliorated, and the Consortium Acute-on-Chronic Liver Failure score decreased by 20 units. The authors conclude that a combined therapy of antibacterial, antifungal, and antiviral therapy with CA-CRRT effectively treats septic shock after LiTx. In conclusion, and also listed in Table 1 (see column “Effects of CA therapy”), many clinical results at different interventional steps (pre-, during, post-) of liver transplantation support the notion that CA therapy may improve the functional graft outcome (↓ bilirubin, ↓ ammonia, ↓ LDH, ↓ platelets), but little evidence is reported regarding changes in early pathological complications (unchanged in-hospital mortality).

### 3.5. Therapeutic Applications in Lymphome and Allogeneic Transplantation

#### Clinical Studies

Stahl applies extracorporeal CA for the treatment of uncontrollable cytokine release syndrome (CRS) [73]. Uncontrollable CRS is a potential life-threatening complication of CD-19 Chimeric antigen receptor-T and is usually treated with IL-6 blockade and steroids. Nonetheless, in this patient, and after CA therapy, they recorded a 50% decrease in multiple soluble inflammatory factors. Despite these promising results, the CA therapy did not reduce the endothelial injury markers, indicating further need for research into the efficacy of the procedure. In a case study, Rademacher provided evidence for the potential efficacy of CA as a supportive treatment for lymphoma-associated hemophagocytic lymphohistiocytosis (HLH) in a patient undergoing allogeneic stem cell transplantation [74]. The patient was able to achieve hematopoietic engraftment and disease-free discharge due to the combination of CA and stem cell transplantation. In conclusion, some clinical evidence in lymphome and allogeneic transplantation support the notion that CA therapy may improve CRS temporarily (↓ cytokines), but little evidences has been reported regarding changes in early pathological complications (unchanged endothelial injury markers).

## 4. Therapeutic Applications of Cytokine Adsorption in Organ Transplantation Complications

### 4.1. Therapeutic Plasmapheresis

#### Clinical Studies

Tomescu makes use of a combination of CA and continuous veno-venous hemofiltration (CVVH), which has the potential to be an effective therapy for SIRS associated with emergency re-transplantation [75]. While CA binds the cytokines, toxins, and mediators, preventing them from circulating in the bloodstream, CVVH works by removing fluid and toxins from the patient’s bloodstream. The combination of HA and CVVH allowed for the rapid and efficient removal of pro- and anti-inflammatory cytokines from the patient’s bloodstream and subsequently renormalized cardiac output and systemic vascular resistance, with rehabilitation of the hepatic function. The combination of these two techniques may prove to be an effective and safe therapy for SIRS associated with emergency re-transplantation. Nakanishi reviews therapeutic plasmapheresis, which has become an important treatment for a variety of diseases that cannot be cured by conventional drug therapy [76]. PE is the most used modality for conditions such as thrombotic microangiopathy and acute hepatic failure. Double-filtration plasmapheresis removes macromolecules, such as immunoglobulins, while avoiding the use of substitution fluids. This is applied in conditions such as hyperviscosity syndrome and ABO-incompatible KTx. Moreover, Salvadori reviews the role of therapeutic apheresis in KTx [77]. Therapeutic apheresis is classified into two main categories: PE and selective apheresis. PE involves the patient’s plasma removal, which is replaced with an appropriate fluid, such as 5% albumin solution, fresh frozen plasma, or a combination of both. Selective apheresis involves the removal of specific blood components or substances, such as red blood cells, platelets, leukocytes, immunoglobulins, lipoproteins, or cytokines, using different techniques, such as centrifugation, filtration, adsorption, or immunoadsorption. Therapeutic apheresis increases the donor graft pool by treating HLA-sensitized patients and making ABO-incompatible KTx possible. Its use in protocols before and after KTx in patients with donor-specific antibodies has a beneficial effect on graft survival and mediates antibody-mediated rejection and the recurrence of primary FSGS. In a case report, Wallet discusses the clinical data of a 79-year-old patient with diffuse aggressive large B-cell lymphoma who relapsed early after first-line treatment [78]. This patient had been treated with a bispecific antibody linking CD3 T cells to CD20 malignant B cells. The patient quickly developed grade 2 CRS, which quickly worsened to grade 4. After excluding a microbiological cause of his condition, this patient received injections of tocilizumab (an antibody against the IL6 receptor). Paradoxically, as tocilizumab can cause a transient increase in IL-6 levels, this can lead to neurotoxicity and an apparent worsening of the situation. In this patient, following 2 days of CA therapy, the overall level of cytokines was decreased. One of the problems often mentioned during CA therapy is the adsorption of drugs (antibiotics, antifungals). However, in this case report, the author notes that the plasma concentrations of these therapeutic agents remained within targeted ranges during the 2 days of CA treatment. In conclusion, some clinical evidence in therapeutical plasmapheresis supports the notion that CA therapy may improve SIRS and may increase the donor graft pool by treating HLA-sensitized patients, making ABO-incompatible KTx possible; however, little evidence, outside of isolated case reports, has been reported regarding changes in early pathological complications.

### 4.2. Endotoxic Septic Shock Complications

Septic shock affects between 5 and 7 million patients each year, and more than half of these patients die [79]. If bacterial toxins or endotoxins are a major cause of septic shock, the appearance of these endotoxins can also be the consequence of other pathologies, such as COVID-19, hence the term endotoxic septic shock. The endotoxic septic shock pathology is characterized by organ failures, such as in the liver and kidneys, as well as vascular system dysfunction caused by disseminated intravascular coagulation or thrombotic microangiopathy. Treatment options for endotoxic septic shock remain limited or are, such as CA therapy, still controversial.

#### 4.2.1. Experimental Studies

Early applications of CA in the management of endotoxic septic shock were proposed by Tetta [80]. The author uses Amberchrome^®^ CG300MD- or Amberlite^®^XAD-1600 (both from Dow Chemical, MI, USA) type resins to completely adsorb certain cytokines, such as IL-Ra, IL-1β, and IL-8. Some of the molecules eluted from the column were characterized with monoclonal antibodies, such as the human alpha2-macroglobulin and human anti-C3c, which demonstrates the capacity of these resins to also eliminate certain cytokines through their indirect binding in association with alpha2-macroglobulin. Moreover, in an experimental approach with rats, Song found that the adsorption rate of the cytokines onto the polymer beads was concentration-dependent and became greater with increasing temperature [81]. The highest adsorption rate observed was at 40 °C, followed by 37 °C and 30 °C. In addition, the kinetics of adsorption of the cytokines onto the beads was independent of calcium concentration. These results suggest that the adsorbent polymer is an effective and efficient adsorbent for various inflammatory cytokines and that temperature and calcium do not significantly affect the adsorption rate. In an early hemofiltration approach in a large animal model, Rimmele discusses the properties of a new hemofiltration membrane modified by a surface treatment, increasing the adsorption of endotoxins [82]. The study compared side by side their proprietary membrane with a control membrane in two models of endotoxic septic shock: a porcine in vivo model and an in vitro contamination model. Regarding the animal model, after 6 h of hemofiltration with the proprietary membrane, the authors demonstrate a reduced need for crystalloids and colloids, a drop in endotoxin concentration, and a normalization of lactic acid levels as well as arterial hypertension compared to the control membrane. They conclude that it is possible to greatly improve the adsorption of endotoxins by treatment of the membranes, consequently ameliorating the hemodynamic levels of pigs.

#### 4.2.2. Clinical Studies

In 2013, Panagiotou reviewed the role of sequential extracorporeal therapies in the treatment of endotoxic septic shock [83], as multiple approaches were developed over time to eliminate cytokines and other CRS modulators in immune system dysregulations, for instance, the development of large-volume hemofiltration membranes with a high threshold coupled with Polymyxin B hemadsorption (PHX-HA)-based resins, or the synergistic development of CRRT with CA therapy. In an early example of human CA therapy, Lees presented a case report of a 33-year-old patient with severe acute respiratory failure with endotoxic and cardiogenic septic shock [84]. This patient subsequently developed ARDS following a bacterial superinfection and a H1N1 pneumonia. To stabilize this patient, they successfully combined a therapy of CA with ECMO that allowed complete recovery. Interestingly, Schadler suggests that CA therapy is not associated with improved outcomes in endotoxic septic shock patients with respiratory failure [85]. The treatment by this author removed IL-6 from patients’ blood, but this transient removal was not enough to cause a sustained decrease of IL-6 levels or to ameliorate the patient outcomes. The study recorded a higher rate of mortality in the treatment group, but this was not statistically significant after adjustment for morbidity and baseline imbalances. Zuccari suggests that HA with CA may be a promising adjunctive therapy for endotoxic septic shock in critically ill adult patients [86]. The findings indicate that it may reduce plasma levels of IL-8 and improve microvascular perfusion, despite no significant variation in macro-hemodynamic parameters. The microcirculation improvement may lead to an overall outcome improvement in septic patients. In a review, De Rosa discusses several important aspects of extracorporeal blood purification therapies to manage endotoxic septic shock and sepsis-associated AKI (SA-AKI) [87]. PMX-HA has been tested in several experimental and clinical studies for treatment of endotoxic septic shock. The results have shown that PMX-HA can effectively reduce endotoxin and cytokine levels in plasma, improve hemodynamic and oxygenation parameters, and decrease mortality rates in endotoxic animals and patients. However, the optimal timing, frequency, and duration of PMX-HA are still unclear, and the long-term effects of PMX-HA on organ function and quality of life are not well established. The clinical indication for PMX-HA is widely debated in the literature. Some experts would initiate PMX-HA as early as possible in patients with suspected or confirmed Gram-negative sepsis and with signs of organ dysfunction or refractory shock. Others would propose PMX-HA for patients with confirmed endotoxemia and with high levels of Endotoxin Activity Assay (EAA), a rapid test that measures the biological activity of endotoxins in whole blood. The EAA is used to monitor the response to PMX-HA and to guide its duration and frequency. Moreover, Ronco reviews the application of extracorporeal therapies for endotoxic septic shock, with the potential to improve outcomes. In particular, the therapy should start after a careful analysis of treatment timing and a careful selection of patients likely to profit from extracorporeal CA therapies [88]. In a 2023 retrospective clinical study, Forin analyzes a cohort of patients suffering from endotoxic septic shock [89]. Of a total of 531 patients included in this study, 61 benefited from at least one hemoperfusion through a column containing immobilized PMX-HA, and 55 of them benefited from a second PMX-HA. However, despite a reduction in the level of EAA and an association of PMX-HA treatment with a functional improvement of affected organs and patient hemodynamics, these data do not reach a significant statistical value. The authors conclude that further randomized trials focusing on patient selection will be necessary to determine when to initiate PMX-HA treatment in patients affected by endotoxic septic shock. In a case report, De Rosa reports the clinical data of a 49-year-old man with bouts of coughing, fever and dysuria that lasted several days following the development of endotoxic septic shock [90]. They obtained a first reduction in plasma endotoxin levels with hemoperfusion through a PMX-HA column and high-volume hemofiltration. On the third day, the patient received a VA-ECMO for 2 days, without improvement, and then CA therapy initiated for 72 h with a veno-venous shunt, again without success. Finally, hemodialysis with a veno-venous shunt including a high cutoff filter was installed, which resulted in an improvement in tachycardial and myocardial functions, with the patient discharged on day 113. This case highlights the importance of sequential extracorporeal therapy when using VA-ECMO in serious cases of endotoxic septic shock. In a 2022 review, Ricci comments on CA therapy as a process that has been studied and applied in the treatment of various medical conditions, such as endotoxic septic shock, AKI, and ALF [91]. The author mentions that recent randomized controlled trials have shown that CA therapy may be beneficial in the treatment of septic shock and MOF as well as being safe and well tolerated by patients. In future development, CA therapy may be associated in combination with other treatments, such as antibiotics. In a 2017 pilot study, Friesecke conducted a trial with 17 patients with refractory septic shock [92]. The author performed hemodynamic measurements and laboratory tests before and after each HP session. The results showed that CA therapy significantly decreased levels of pro-inflammatory cytokines and improved hemodynamic parameters such as MAP and cardiac index. Moreover, the study revealed a decrease in markers of oxidative stress, suggesting that the technique may have beneficial effects on the metabolic aspects of septic shock. In a cases series, Kogelmann evaluated CA therapy in 26 patients with endotoxic septic shock and requiring CRRT [93]. In the early-treated patients, the author observed a stabilization of hemodynamic factors as well as a drop in lactic acid levels. It was noted, however, that any delay in the initiation of CA therapy (beyond 24 h after a diagnosis of endotoxic septic shock) led to a drop in the level of positive response to treatment. CA treatment was well tolerated, as no adverse effects were observed. In a follow-up study, Kogelmann showed that the septic shock Dynamic Scoring System (DSS) helped to identify patients that would present a high risk of mortality, i.e., with a DSS score > 4.4, and associated with a mortality rate of >30%, indicating a clear cut-off for the initiation of adjunctive CA therapy [94]. Furthermore, a delay of more than 12 h between the onset of septic shock and the start of CA therapy was linked with a bad outcome with regard to mortality and hospital stay. This suggests that the earlier a patient is treated, the better the outcome. These findings support the use of the DSS score as a tool to sort patients with refractory septic shock who might profit from adjunctive CA treatment. In a case report, David presents the clinical data of a 32-year-old female with significant endotoxic septic shock accompanied by AKI [95]. Since the patient remained hypotensive and unresponsive to SMT, the author initiated CA therapy. The patient’s blood having been collected before and after CA therapy, this serum was tested, in vitro, on a model with human umbilical vein cells. This analysis showed that intercellular contacts were altered, which led to functional alterations in membrane permeability. These deleterious effects were different in contact with the serum treated with CA therapy. In this case, the cells were conversely protected from the harmful effects of the cytokine cascade. In another case report, Bruenger presents a 70-year-old male patient with severe sepsis due to Staphylococcus aureus bacteremia, complicated by ARDS, septic shock, and MOF [96]. The patient had been admitted to the ICU and was initially stabilized using conventional supportive care, including antibiotics, fluids, and vasopressors. However, his condition deteriorated, and he developed severe hypotension, increased lactate levels, and metabolic acidosis. The team started left ventricular assist device support, ECMO, CVVH, and CA. The patient responded well to the combined therapy, with improved organ perfusion, normalization of lactate levels, and resolution of metabolic acidosis. The patient was weaned off ECMO and left ventricular assist device support and subsequently discharged from the hospital in good condition. This case report demonstrates that the combined use of left ventricular assist device, ECMO, CVVH, and CA is a viable option for management of severe endotoxic septic shock in critically ill patients. The combination of devices could be a reasonable approach for the management of sepsis in patients with multiple organ dysfunction, especially when conventional supportive care is insufficient. In a large animal model of smoke-induced lung damage and deep burn injuries, Linden studied CA therapy in the 72 h following injuries, for a total of three sessions of 6 h [97]. Despite significant removal of circulating myoglobins and cytokines (IL-1β, IL6, IL-10) across the CA device, the author reports that CA therapy did not decrease the systemic or pulmonary levels of these molecules. In 2019, Netti reported both data from a clinical trial of CPFA therapy in patients with septic shock and data from PMX-HA with a porcine LPS-induced sepsis model [98]. In both types of experiences, the levels of cytokines, CRP, procalcitonin, and endotoxins decreased significantly. As proteinuria, albuminuria, and CD80 levels also decreased, the authors hypothesize that the podocytes were protected by a reduction in CD80 overexpression, which led to a general improvement in their condition and a reduction of the risk of progressive chronic renal failure. In a case report, Li presents the clinical data of a 35-year-old man with hepatitis B who suffered septic shock after liver transplantation [53]. The oXiris^®^ (Baxter International Inc., Deerfield, IL USA) hemofilter incorporated into CVVH was proposed in addition to CRRT treatment with antifungal, antiviral, and antibacterial agents. After 48 h of oXiris^®^ (Baxter International Inc., Deerfield, IL USA) hemofilter treatment, blood pressure was stabilized as well as renal and hepatic functions, while certain inflammatory modulators, such as IL-6 and IL-10, fell sharply. In a series of reviews, Ankawi [99] [100], Monard [101], and De Rosa [102] comment on several important aspects and different systems of extracorporeal blood purification therapies to manage endotoxic septic shock and organ dysfunctions. They provide sample cases to consider CA as a potential therapeutic option and list the demonstrated potential efficacy and safety in the treatment of acute inflammatory conditions. In a single-center retrospective study, Rugg demonstrates the potential of CA to improve hemodynamics and metabolism in refractory septic shock [103]. The study analyzes septic shock patients who received CA therapy with CytoSorb^®^ (CytoSorbents, Monmouth Junction, NJ, USA) in addition to CCRT. This CA therapy cohort is compared to a matched control group without CA therapy. Baseline comparabilities between the two groups were high, with differences primarily in higher initial SOFA scores and norepinephrine equivalent requirements in the CA therapy group. Catecholamine use and in-hospital mortality decreased in the CA therapy group compared to the control group. The author reports that lactate levels are predictive of mortality, with high specificity. In a cohort of patients, Mariano tests the therapies with or without CPFA in patients who have suffered from severe burns, endotoxic septic shock, and AKI [104]. The mortality rate significantly decreased for the group of 39 patients treated with CPFA (51.3%) compared to the 87 patients treated without CPFA (77.1%). This study also accumulated data concerning the safety of CPFA therapy (i.e., bleeding incidents, difficulties associated with the catheter, and hypersensitivity reactions) but also with respect to the possible improvement in the effectiveness of CPFA (frequency and duration of CPFA therapies, as well as quantity of plasma processed during these CPFA sessions). In conclusion, some clinical and experimental evidence in the management of endotoxic shock complications supports the notion that CA therapy may improve functional organ outcomes (↓ proteinuria, ↓ endotoxins in plasma, ↑ hemodynamic, ↑ oxygenation), with some evidence reported regarding changes in early pathological complications (↓ in-hospital mortality, ↓ mortality rates in endotoxic animals).

### 4.3. Poisoning Complications and Removal of Pharmaceuticals

#### 4.3.1. Experimental Studies

In an experimental setting, Godi determines the adsorption capacity of the HA380 cartridge (HA380, Jafron Biomedical, Zhuhai City, China) towards the antibiotic vancomycin [105]. Vancomycin is a glycopeptide antibiotic commonly used to treat infections caused by Gram-positive bacteria. The study recorded a rapid and potent decrease in concentration until reaching a plateau. About 90% of the 5000 mg of vancomycin adsorption occurred in the first 40 min. At a higher concentration of 10,000 mg vancomycin, the one-hour adsorption efficiency dropped to 55%. These findings suggest that the HA380 cartridge has a significant vancomycin adsorption capacity, which could have implications for its use in clinical settings. Moreover, Lorenzin also determined the amount of vancomycin adsorbed by a HA380 cartridge (HA380, Jafron Biomedical, Zhuhai City, China). Vancomycin use is often associated with adverse side effects, such as nephrotoxicity, ototoxicity, and the development of antibiotic-resistant strains [106]. The experiments were carried out as in Godi [105], and with 10,000 mg vancomycin, the cartridge also plateaued after 60 min. Finally, De Cal discusses the application of the HA380 cartridge (HA380, Jafron Biomedical, Zhuhai City, China) in extracorporeal therapy for sepsis treatment [107]. The study evaluates the effect of bacteria and vancomycin removal by CA and its interaction with antibiotic therapy. The study was conducted in vitro and found that the absorbent cartridge was effective in reducing bacterial concentration in blood samples.

#### 4.3.2. Clinical Studies

In a meta-analysis, Hawchar reviews the literature and indicates that CA is effective in reducing norepinephrine requirements in critically ill patients [108]. The pooled effect size from the 33 studies selected in the literature and after one day of CA therapy was large and significant but indicated a high level of diversity with regard to the extent of the treatment outcome. In a case report, Giuntoli [109] presents the clinical data of a female patient with unknown anamnesis and brought to the emergency room after voluntary quetiapine (Seroquel^®^) intake. The patient reported that she had taken 10 tablets of quetiapine, each containing 25 mg of quetiapine fumarate. Given the patient’s mental status and the presence of a prolonged corrected QT interval, she was intubated and admitted to the ICU. To accelerate quetiapine elimination, they decided to employ CA therapy in combination with CRRT. After the CA procedure, the patient was extubated the following day and recovered uneventfully. The combination of CA and CRRT resulted in a rapid reduction of the quetiapine plasma concentration, allowing the patient’s mental status and ECG parameters to improve. In another case report, Dalmastri [110] presents the successful treatment of a 82-year-old Caucasian male with AKI who was chronically anticoagulated with apixaban therapy. Apixaban is a non-vitamin K antagonist oral anticoagulant that inhibits factor Xa. As the literature reported that other antithrombotic agents such as rivaroxaban and ticagrelor could be successfully eliminated by CA therapy, this option was chosen for this patient, treated with apixaban but now awaiting urgently a nephrostomy. CCRT coupled with CA therapy quickly neutralized apixaban, which made it possible to organize the surgical intervention without postoperative complications. In a case report, Koster used CA effectively during HTx to remove argatroban, a thrombin inhibitor to manage heparin-induced thrombocytopenia [37]. This strategy allowed a rapid reduction of argatroban concentrations and helped to achieve satisfactory hemostasis. In a single-center retrospective study, Scandroglio reports that CA treatment did not significantly decrease the dose requirement of vancomycin (antistaphylococcal antibiotic) or bivalirudin (an anticoagulant) in the critically ill patients studied [111]. The study concluded that CA is a secure and potent procedure for afflicted patients in critical conditions and does not considerably affect the dose requirements of vancomycin or bivalirudin. Both medications have been shown to be effective in preventing clot formation and in improving outcomes in patients undergoing cardiovascular procedures. In conclusion, some clinical and experimental evidence in the management of poisoning complications and removal of pharmaceuticals supports the notion that CA therapy may improve functional organ outcomes (↓ antibiotics, ↓ anticoagulants, ↑ hemostasis), with rare evidence out of some case reports regarding changes in early pathological complications.

## 5. Conclusions

A recent and promising therapeutic approach in transplantation surgery is the increasing application of cytokine adsorption methods, carried out either during the surgical procedure or before surgery with ex vivo graft treatment and even applied all along those procedures. Convincing research to justify hemoadsorption during the post-surgical phase is, at this stage, only in its infancy. Many physiological parameters are involved during hemoadsorption and remain insufficiently documented. To improve the quality and applicability of approaches combining hemoadsorption with organ transplantation procedures, further large-scale, carefully designed and monitored clinical trials remain essential.

## Data Availability

Not applicable.

## Abbreviations

ARDS	acute respiratory distress syndrome
AKI	acute kidney injury
ALF	acute liver failure
ALI	acute lung injury
ACLF	acute-on-chronic liver failure
APACHE	acute physiology and chronic health evaluation
BP	blood perfusion
CA	cytokine adsorption
CRS	cytokine release syndrome
CPB	cardiopulmonary bypass
CTA	cellulose triacetate
CRRT	continuous renal replacement therapy
CVVH	continuous veno-venous hemofiltration
CPFA	coupled plasma filtration and adsorption
CRP	C-reactive protein
DCD	donation after circulatory death
DPMAS	Double Plasma Molecular Adsorption System
EAA	Endotoxin Activity Assay
ECMO	extracorporeal membrane oxygenation
EBP	extracorporeal blood purification therapies
EVLP	ex vivo lung perfusion
FSGS	focal segmental glomerulosclerosis
HTx	heart transplantation
HD	hemodialysis
HP	hemoperfusion
HA	hemoadsorption
IL	interleukins
ICU	intensive care unit
IRI	ischemia reperfusion injury
KTx	kidney transplantation
LiTx	liver transplantation
LuTx	lung transplantation
MARS®	Molecular Adsorbent Recycling System
MAP	mean arterial pressure
MCO	medium cut-off
MOF	multiple organ failure
NMP	normothermic machine perfusion
PE	plasma exchange
PMMA	polymethyl methacrylate
PMX-HA	polymyxin B hemadsorption
PAES: PVP	polyvinylpyrrolidone: polyarylethersulfone
PGD	primary graft dysfunction
SA-AKI	sepsis-associated AKI
SIRS	severe inflammatory response syndrome
SOFA	Sequential Organ Failure Assessment
SMT	standard medical therapy
TNF-α	tumor necrosis factor alpha
VA-ECMO	veno-arterial extracorporeal membrane oxygenation
